# Simultaneous Transcatheter Aortic and Mitral Native Valve Replacement: A Step-by-Step Procedural Approach

**DOI:** 10.1016/j.shj.2024.100295

**Published:** 2024-03-13

**Authors:** Julio I. Farjat-Pasos, Dimitri Kalavrouziotis, Jonathan Beaudoin, Jean-Michel Paradis

**Affiliations:** a Department of Structural Interventional Cardiology, Quebec Heart and Lung Institute, Quebec City, Quebec, Canada; b Department of Cardiac Surgery, Quebec Heart and Lung Institute, Quebec City, Quebec, Canada; c Department of Echocardiography, Quebec Heart and Lung Institute, Quebec City, Quebec, Canada

**Keywords:** Aortic regurgitation, Mitral stenosis, Multivalvular heart disease, Simultaneous transcatheter valve replacement, Transcatheter aortic valve replacement, Transcatheter mitral valve replacement

## Abstract

Multivalvular heart disease (MVHD) is present in one-third of patients with valvular heart disease (VHD). Compared to single VHD patients, these patients have a more significant hemodynamic impact and are often left under medical treatment. Most importantly, when undergoing multiple valve interventions, they show worse rates of heart failure and mortality. The guidelines-supported interventions in patients with MVHD in combined aortic regurgitation and mitral stenosis include percutaneous mitral balloon commissurotomy, open mitral commissurotomy, or surgical mitral valve replacement followed by transcatheter or surgical aortic valve replacement, trying to minimize the increased mortality risk of double-valve replacement. Simultaneous transcatheter valve replacement (STVR) for native MVHD is still off-label and not yet considered in clinical guidelines since the evidence of its results is limited to a few cases reported worldwide. However, fully percutaneous transfemoral STVR seems promising for MVHD patients thanks to its minimal invasiveness, the continuous improvement of the transcatheter heart valve devices, the likely shorter length of stay and the fastest recovery. To our knowledge, this is the first case ever reported of fully percutaneous STVR for native MVHD in aortic regurgitation and mitral stenosis. Deep understanding of both pathologies and their interactions, not only from a pathological point of view but from the procedural planning and procedural steps point of view is mandatory. Hereby we present the specific STVR procedural planning considerations, a step-by-step guide on how to perform an aortic and mitral STVR and its critical considerations, as well as the procedural and follow-up results.

## Introduction

Multiple valvular heart disease (MVHD) is a highly prevalent condition.^1^ Patients with MVHD have a more significant hemodynamic impact and are more complex to evaluate since there are no specific risk-estimating tools for these patients.^2^ Some patients suffering from MVHD are not infrequently left under medical treatment since they are judged nonoperable due to their estimated prohibited or very-high surgical risk.^3^ Moreover, recommendations of major clinical management guidelines on patients with valvular heart disease (VHD) are scarce given the lack of data and complexity of this particular subgroup of patients.^4^^,^^5^ Additionally, these patients show worse heart failure and mortality rates after valve interventions than patients with single-valve disease.^3^

Radiation-induced cardiovascular disease can result in various manifestations, including coronary artery disease, cardiomyopathy, conduction system abnormalities, pericardial disease, and VHD, with left-sided valves most commonly affected despite the anterior position of the right heart structures.^6^^,^^7^ Nevertheless, when it comes to surgical valve replacement, patients with a history of mediastinal radiation have worse short- and long-term outcomes, likely due to a combination of the need for concurrent cardiac procedures, higher rates of conduction disturbances requiring device therapy, and postoperative ventricular dysfunction, which is even worse in the presence of MVHD.^8^^,^^9^

The guidelines-supported interventions in patients with MVHD in combined aortic regurgitation (AR) and mitral stenosis (MS) depend on the mitral anatomy. Options include percutaneous mitral balloon commissurotomy, open mitral commissurotomy,^4^^,^^5^ or surgical mitral valve replacement followed by transcatheter or surgical aortic valve replacement, trying to minimize the increased mortality risk of double-valve replacement.^10^

Simultaneous transcatheter valve replacement (STVR) for multivalvular aortic and mitral valve disease is still off-label and not yet considered in clinical guidelines since the evidence of its results is limited to a few cases reported worldwide.^11^ However, fully percutaneous transfemoral STVR seems promising for MVHD patients thanks to its minimal invasiveness, the continuous improvement of the transcatheter heart valve (THV) devices, the likely shorter length of stay and the fastest recovery.^11^

To this day, there are reports on only 3 cases of fully percutaneous transfemoral STVR for patients with MVHD with aortic stenosis and MS^12^, ^13^, ^14^; however, fully-percutaneous transfemoral STVR in patients with AR and MS has never been reported. In combined AR and MS, MS is usually the more severe lesion.^15^ Because MS limits left ventricle (LV) filling, it may reduce the stroke volume presented to the aortic valve, reducing the apparent severity of AR.^16^ Furthermore, MS reduces the LV cavity size for any degree of AR, causing further potential underestimation of AR severity.^15^^,^^16^ From a transcatheter procedural perspective, several additional considerations impose a more significant challenge than a combined STVR for aortic stenosis and MS.

Hereby we describe a case of a patient with combined AR and MS on which we performed a fully percutaneous transfemoral STVR. We describe the preprocedural planning, the step-by-step guide for the procedure, the critical technical considerations, and the follow-up results.

## Case Presentation

This was a case of a 66-year-old female patient with a history of hypertension, dyslipidemia, and left bundle branch block. In 1976, she had Hodgkin's lymphoma, which was treated with chemotherapy and high-dose radiation therapy resulting in secondary hypothyroidism and postradiation arteriopathy with severe porcelain aorta. Her ambulatory medical treatment consisted of aspirin, atorvastatin, bisoprolol, furosemide, and spironolactone.

She developed progressive shortness of breath and a New York Heart Association functional class III over 6 ​months. The echocardiographic evaluation showed a LV ejection fraction of 43% with severe mitral annulus calcification (MAC) and severe MS (mitral valve max/mean gradients 31/10 mmHg, mitral valve area 0.95 cm^2^) with concomitant moderate-to-severe AR with no aortic stenosis (aortic valve max/mean gradients 27/11 mmHg). The estimated systolic pulmonary arterial pressure was 63 mmHg with a dilated right ventricle, although its systolic function was preserved. The coronary angiography showed nonobstructive coronary artery disease. After a lack of response to medical treatment optimization, the heart team assessed the possibility of double native valve replacement. Patient’s Society of Thoracic Surgeons scores for surgical mitral and aortic valve replacements were 7.1 and 4.5%, respectively. The patient was deemed not a candidate for surgery because of the porcelain aorta and the high calcium burden on the mitral valve. Therefore, an STVR of native aortic and mitral valves was considered. After discussing the risks and benefits with the patient and signing informed consent, the patient agreed to be evaluated for procedural feasibility.

## Preprocedural Evaluation

Transcatheter Aortic Valve Replacement

**Figure 1:**
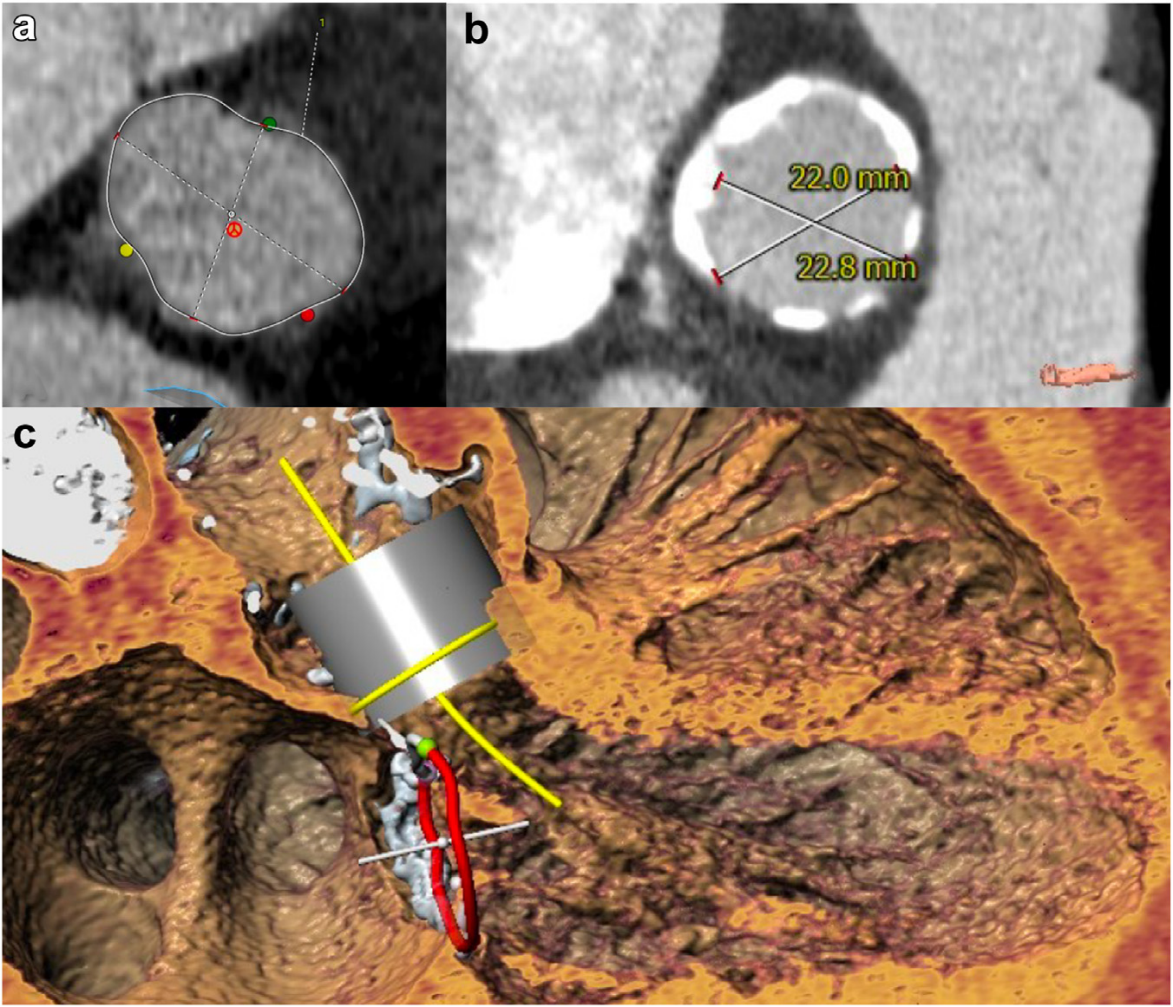
**Transcatheter aortic valve replacementplanning.** (a) Aortic valve annulus segmentation (green dot – right sinus; red dot – left sinus; yellow dot – noncoronary sinus) showing no annular calcification. (b) STJ showing severe circumferential calcification (porcelain aorta) with a restrictive diameter for any valve above 20 mm. (c) Simulated 23 mm SAPIEN S3 Ultra (Edwards Lifesciences, California, USA) valve in aortic position with depth estimation related to the aortic annulus (yellow circle) to avoid the STJ but without a deep implant to avoid interaction with the mitral annulus (red circle). Reconstruction made with 3Mensio (Pie Medical, Maastricht, the Netherlands) Structural Aortic Valve package. Abbreviation: STJ, sinotubular junction.

- Transcatheter heart valve in AR: The computed tomography showed a nonhorizontal hostile, severely calcified ascending aorta with a sinotubular junction diameter of 22 mm, an aortic valve area of 346 mm^2^ with a mean diameter of 20.8 mm and a perimeter of 67 mm. There was no calcium at the aortic valve annulus level. The valve was a trileaflet valve with mild calcification at the tip of the leaflets (total Ca score of the aortic valve: 466 AU). Left and right coronary heights were 10 mm each, and the arterial iliofemoral axes were above 5.5 mm (Figure 1).

Transcatheter Mitral Valve Replacement

**Figure 2:**
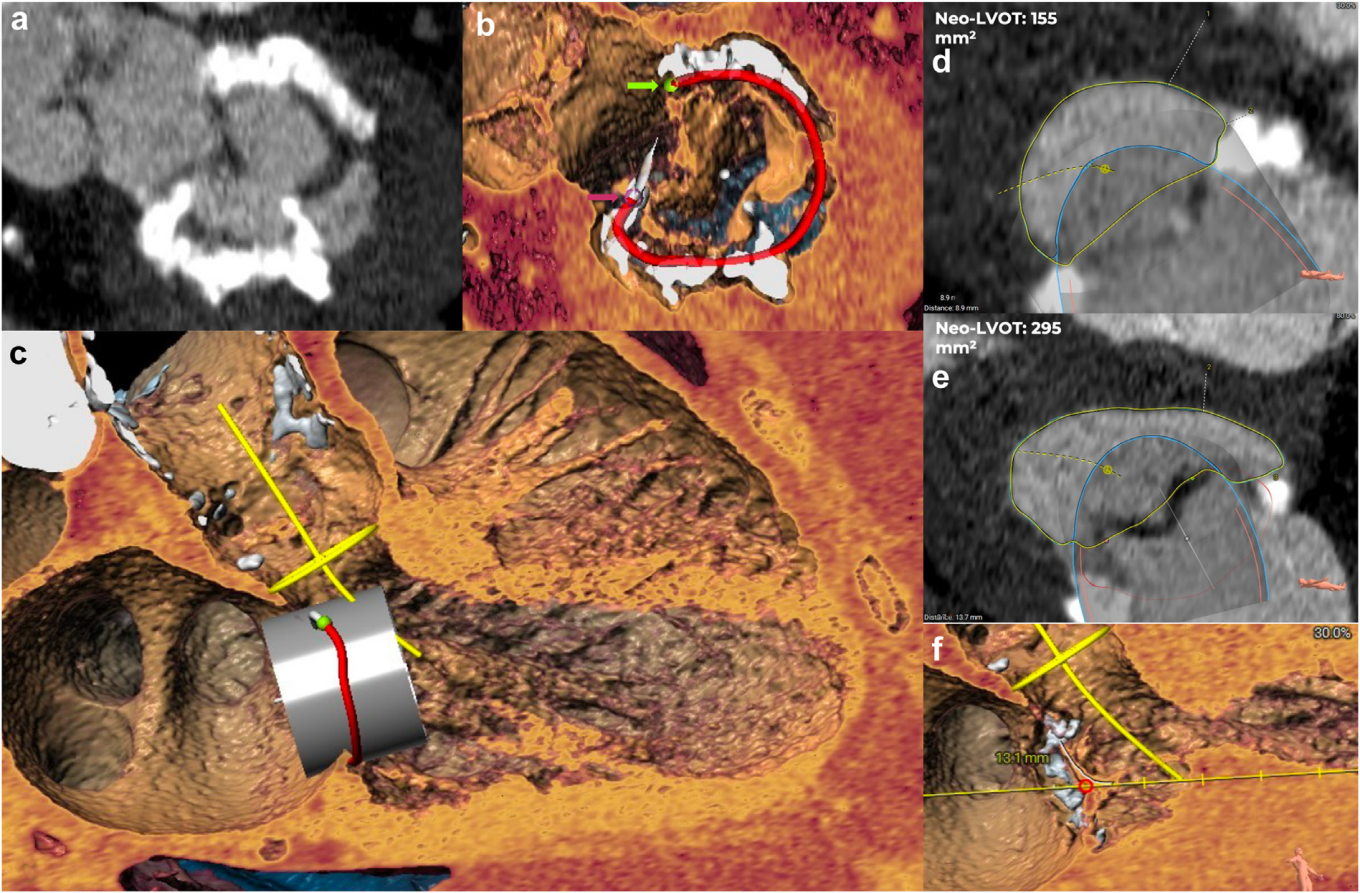
**Transcatheter mitral valve replacementplanning.** (a) Mitral valve calcium distribution with a mitral annulus calcification score of 8 points. (b) Mitral annulus segmentation with a D shape (red demi-circle) showing both trigones (green and red dots, pointed out by the arrows). (c) Simulated 26-mm SAPIEN S3 Ultra (Edwards Lifesciences, California, USA) valve in mitral position with depth estimation related to the mitral annulus (red circle) to avoid interaction with the LVOT and to show its distance in relation to the aortic annular plane (yellow line) and its center line from the ascending aorta to the center of the LVOT (yellow line). (d) Systolic neo-LVOT measurements. (e) Diastolic neo-LVOT measurements. (f) Measurement of the anterior leaflet of the mitral valve. Reconstruction made with 3Mensio (Pie Medical, Maastricht, the Netherlands) Structural Mitral Valve package. Abbreviation: LVOT, left ventricle outflow tract.

- Transcatheter heart valve in MAC: The computed tomography showed a normal interatrial septum, a moderate to severe MAC with a MAC score of 8 points, thus, a low risk of embolization, an aortomitral angle of 70º and an annulus-center to apex length of 81 mm. The mitral annulus area was 480 mm^2^ with a perimeter of 82 mm. The trigone-to-trigone distance was 17 mm, the septolateral distance was 25 mm, and the anterior-to-posterior distance was 22 mm. With a virtual 26 mm SAPIEN S3 Ultra (Edwards Lifesciences, California, USA) THV, the diastolic neo-LV outflow tract (LVOT) was 295 mm^2^ and in systole, 155 mm^2^. Thus, the risk of LVOT obstruction (LVOTO) was not neglectable. However, the interventricular septum had 9 mm thickness, and the mitral valve anterior leaflet was 13 mm long (Figure 2); thus, no leaflet (e.g., laceration of the anterior mitral leaflet to prevent LVOTO) or septal (alcohol septal ablation) transcatheter modification techniques were considered.

## Procedural Description

### Obtaining All Vascular Accesses

**Figure 3:**
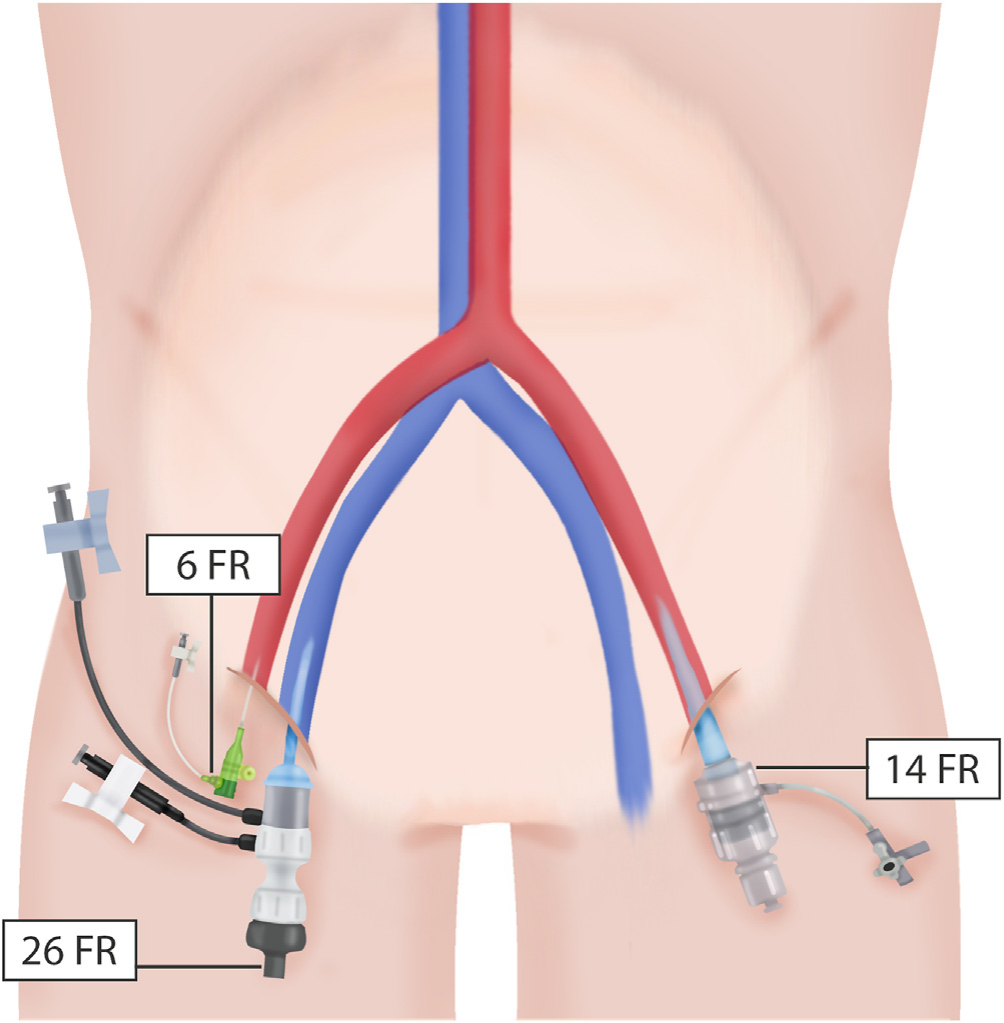
**Vascular accesses configuration for simultaneous transcatheter aortic and mitral native valve replacement (STVR).** Vascular accesses configuration for simultaneous transcatheter aortic and mitral valve replacement (STVR). Starting with the main transcatheter mitral valve replacement access in the femoral vein, placing a 6-Fr introducer. This will be eventually upgraded to a 26-Fr × 65 cm GORE DrySeal Flex Introducer Sheath (Gore Medical, Delaware, USA). The second access to obtain is the secondary transcatheter aortic valve replacement (TAVR) access, which will be placed into the right femoral artery using a 6-Fr introducer sheath that will be used for aortograms but also for crossing over to the contralateral femoral artery. The third access is the main TAVR access in the left femoral artery using a 6-Fr catheter that will be eventually upgraded to a 9-Fr and finally to the 14-Fr eSheath (Edwards Lifesciences, California, USA).

The first step of the simultaneous multivalvular transcatheter replacement was obtaining all vascular accesses with staged heparinization. First is the venous access. The right femoral vein for the transcatheter mitral valve replacement (TMVR) procedure and the left artery for the transcatheter aortic valve replacement (TAVR) since this would allow standard equipment angulation for the TMVR procedure. For both accesses, we used a preclosure technique with 2 Perclose ProStyle devices (Abbott, Chicago, USA). The femoral artery access was obtained with a cross-over technique, an alternative vascular access management strategy that can ensure percutaneous treatment of potential vascular complications of the main TAVR left femoral access. At this point, we administered half a dose of heparin (50 U/kg) (Figure 3).

### Setting Both Procedures

**Figure 4:**
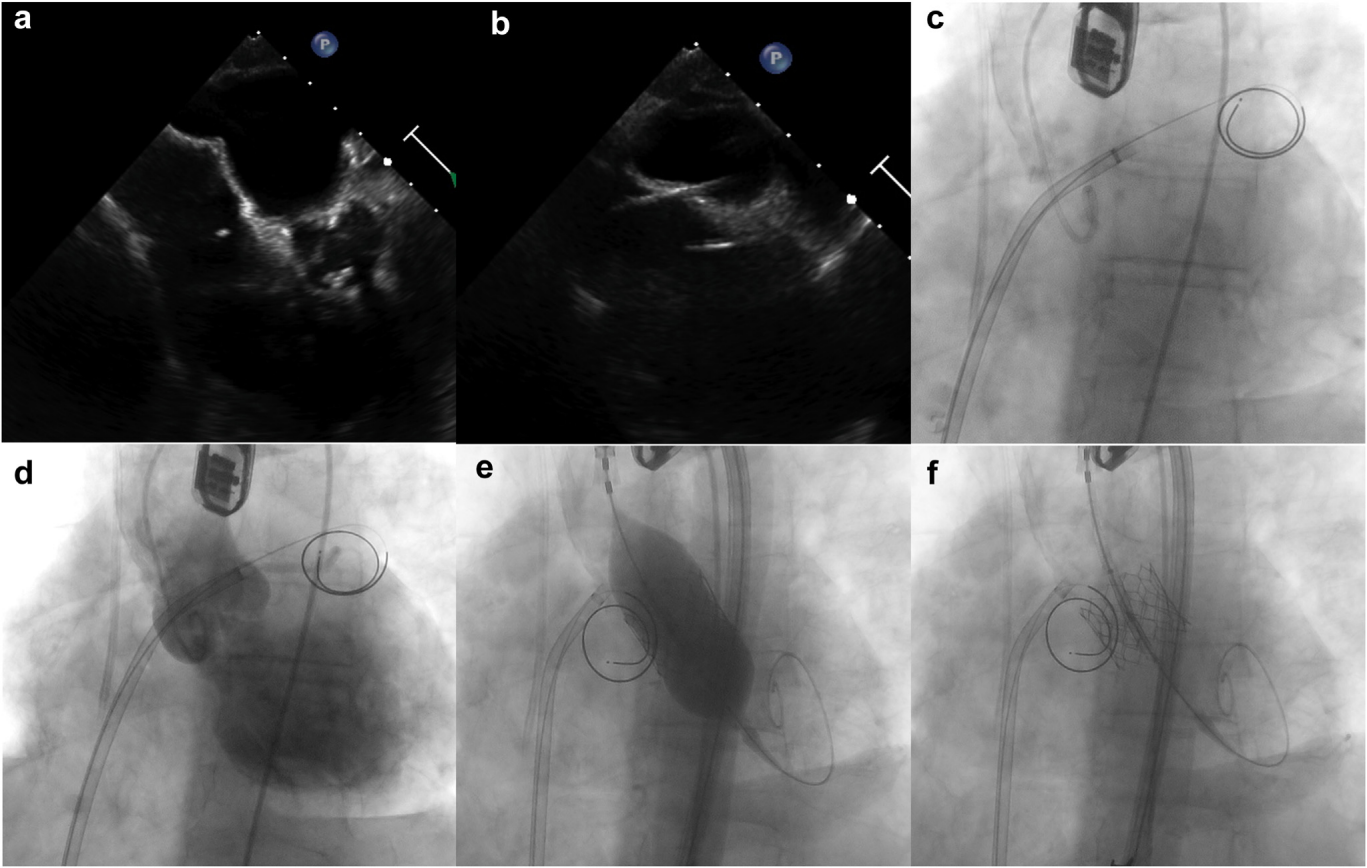
**Transeptal puncture and transcatheter aortic valve replacement.** (a and b) Transesophageal echocardiography guidance aiming for a posterior and inferior puncture to access the LA. (c) After the transeptal puncture, the VersaCross RF System (Baylis Medical, Montreal, Canada) and the VersaCross RF Pigtail wire (Baylis Medical, Montreal, Canada) were placed in the LA. (d) Aortography confirmed severe aortic regurgitation. (e) Transcatheter aortic valve replacement under rapid pacing via the left ventricle wire. (f) The 23-mm SAPIEN S3 Ultra (Edwards Lifesciences, California, USA) transcatheter heart valve deployed in the aortic position. Abbreviation: LA, left atrium.

The next step was the transeptal puncture (Supplemental Video 1). At this point, we exchanged the right venous introducer for a VersaCross RF System (Baylis Medical, Montreal, Canada). Under transesophageal echocardiographic guidance, we performed the transeptal puncture using a VersaCross RF Pigtail wire (Baylis Medical, Montreal, Canada). We secured the VersaCross RF System and the wire into the left atrium to ensure future access to it for the TMVR procedure. After excluding any pericardial effusion, the patient was fully heparinized (another 50 U/Kg for a total of 100 U/kg), aiming for an activated clotting time of 250 (Figure 4).

### Transcatheter Aortic Valve Replacement for AR

The 3-cusp aortogram confirmed severe AR (Supplemental Video 2). After securing LV access with a Safari^2^ wire (Boston Scientific, Massachusetts, USA), we exchanged the 9-Fr arterial introducer for the 14-Fr eSheath (Edwards Lifesciences, California, USA). Based on the aortic valve area and the desired 20% oversize rate for AR, a 23-mm SAPIEN S3 Ultra (Edwards Lifesciences, California, USA) valve was selected, which we deployed under rapid pacing (180 per minute) using the LV wire (Figure 4 and Supplemental Video 3). A critical technical detail was to do slightly slower valve inflation since, in patients with severe AR, the risk of missing the desired landing zone is higher than in patients with aortic stenosis; thus, the potential need for acute valve position adjustment during deployment is not uncommon. An important consideration when performing an aortic-mitral STRV is keeping the LV wire in place after aortic valve deployment and even after the TMVR procedure. By doing so, the wire can be safely used for LV pacing while performing the TMVR, and it can also be used to treat LVOTO in a bailout scenario after the TMVR procedure.

### TMVR: Valve-in-MAC

**Figure 5:**
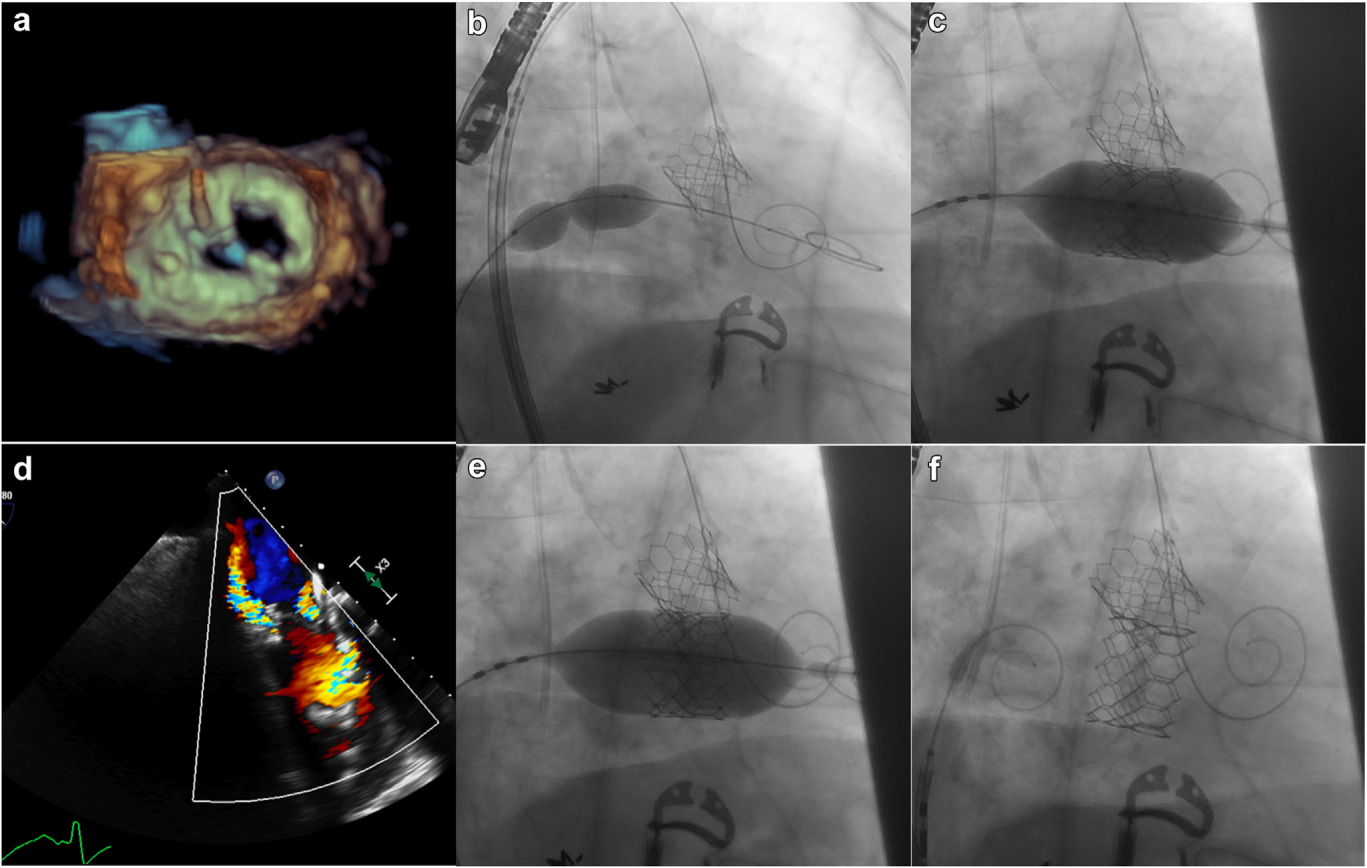
**Mitral valve safe crossing, predilation and mitral valve deployment.** (a) 3D TEEmitral valve reconstruction to guide the crossing of the mitral valve to get access to the left ventricle (LV). (b) Interatrial septum predilation with a 40 × 16 mm Z-Med balloon. This picture also demonstrates that the Safari^2^ wire (Boston Scientific, Massachusetts, USA) across the aortic valve was voluntarily left in place to be able to intervene (kissing balloon inflation) in case of LVOT obstruction after TMVR. (c) TMVR under rapid pacing through the LV wire. (d). TEE showing a moderate paravalvular leak with no LVOT obstruction. (e) Postdilation of the 26 mm SAPIEN S3 Ultra valve (Edwards Lifesciences, California, USA) with 3 mL extra. (f) Final fluoroscopic result of the simultaneous transcatheter aortic and mitral replacements for native multivalvular heart disease. Abbreviations: LVOT, left ventricular outflow tract; TEE, transesophageal echocardiography, TMVR, transcatheter mitral valve replacement.

The next step was the interchange of the VersaCross RF System (Baylis Medical, Montreal, Canada) for a medium Agilis NxT Steerable Introducer (Abbott, Chicago, USA) to guide mitral valve crossing under 3-dimensional echo (Supplemental Video 4). Once the pigtail had crossed, we positioned a second Safari^2^ wire at the LV apex. This step should be done slowly to prevent catheter prolapse in the left atrium (Figure 5) and while keeping the curve of the Safari^2^ wire facing the upper part of the LV to maintain coaxiality of the delivery system through the wire.

After vein predilation, we exchanged for a 26 Fr × 65 cm GORE DrySeal Flex Introducer Sheath (Gore Medical, Delaware, USA). The use of this specific sheath is very helpful, particularly when trying to combine 2 procedures, since it allows the valve to be crimped directly on the balloon, as opposed to using the eSheath (Edwards Lifesciences, California, USA), where the balloon needs to be retracted into the crimped transcatheter heart valve within the inferior vena cava. We used the same 4.0 cm × 16 mm Z-MED (NuMED Inc, New York, USA) balloon to sequentially predilate the mitral valve (nominal full inflation) and then the interatrial septum (using gentle partial inflation) (Supplemental Video 5). Using the Commander (Edwards Lifesciences, California, USA) delivery system, a 26-mm SAPIEN S3 Ultra (Edwards Lifesciences, California, USA) THV with an additional 1 ml was deployed under rapid pacing (180 per minute) through the LV wire (Supplemental Video 6). A critical point here is to inflate slowly to adjust the valve position while it is being deployed. The immediate transesophageal echocardiographic evaluation showed a moderate paravalvular leak with no signs of LVOTO (Supplemental Video 7). The delivery system balloon was reinflated with an extra 3 mL (Supplemental Video 8), significantly reducing the paravalvular leak to trace (Figure 5 and Supplemental Video 9).

### Vascular Closure

**Figure 6:**
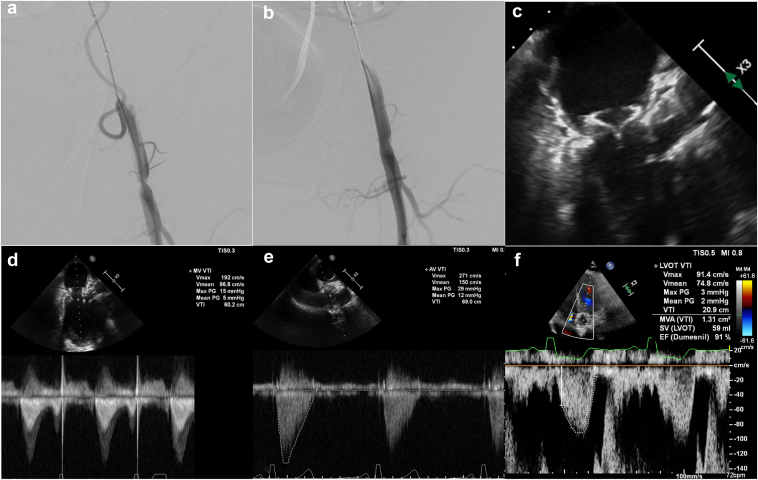
**Vascular access management, procedural and follow-up results.** (a) Angiography of the main transcatheter aortic valve replacement access via the cross-over balloon showing significant residual stenosis after vascular closure devices were closed. (b) Result after balloon angioplasty using the same premounted over-the-wire balloon from the contralateral femoral artery. (c–e) Postprocedural TEEevaluation showing: c – good valves position and function with no mechanical obstruction of the LVOT; d – mean mitral valve gradient of 5 mmHg; e – mean aortic valve gradient of 12 mmHg. (f) One-month follow-up transthoracic echocardiogram still showing no signs of LVOT obstruction. Abbreviations: LVOT, left ventricule outflow tract; TEE, transesophageal echocardiography.

**Figure 7:**
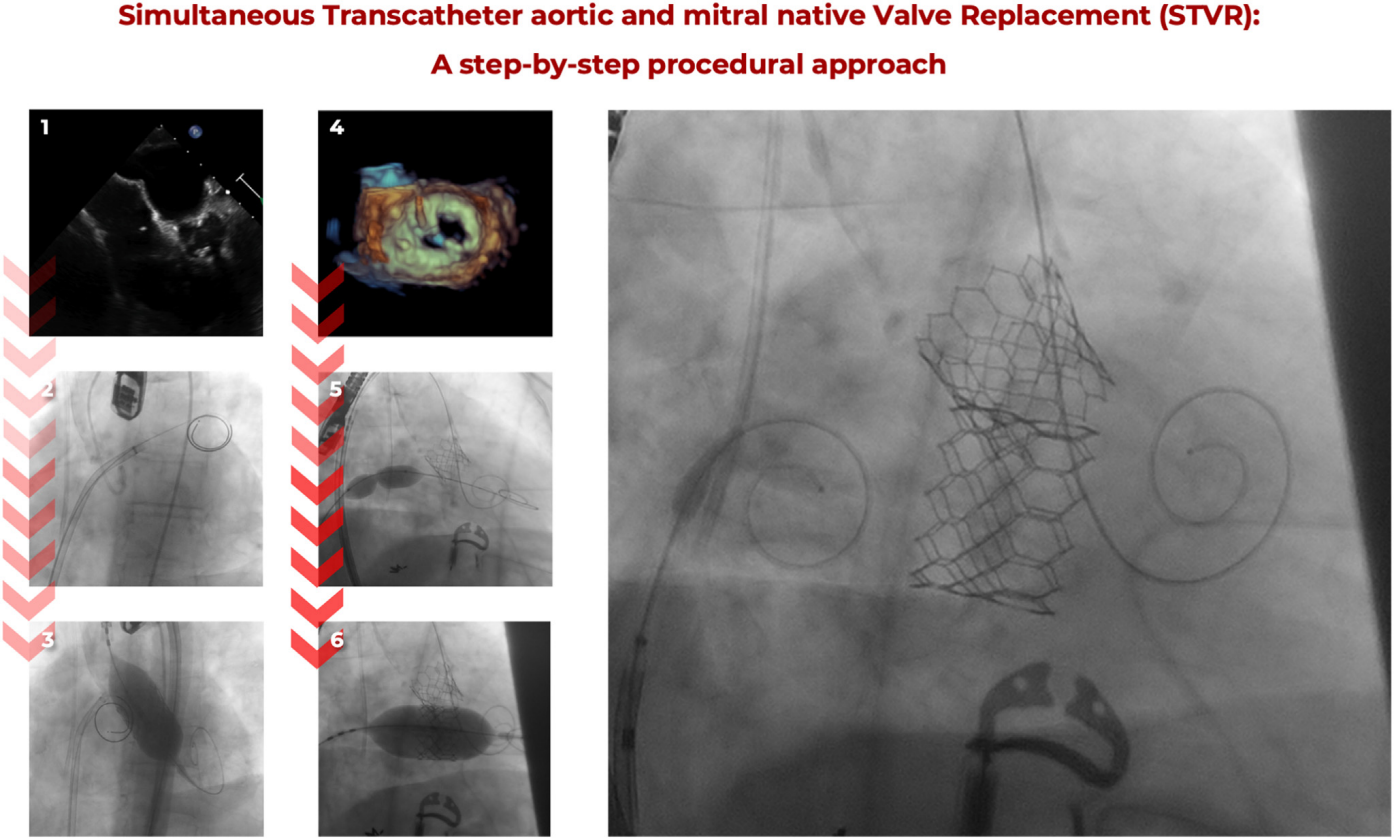
**Simultaneous Transcatheter aortic and mitral native valve replacement (STVR).** After setting vascular accesses (main TAVR-main access into the left femoral artery, main TMVR-main access into the right femoral vein and secondary TAVR access into the right femoral artery) and after giving half-dose heparin, the first STVR procedural step (1) is performing the TEE-guided transeptal puncture and then complete the heparin dose. Leaving the transeptal puncture system in place, the pre-TAVR aortogram is performed (2) to finally deploy the aortic THV (3) while holding the LV wire into the LV after the aortic THV deployment. Under TEE guidance, the native mitral valve is crossed (4) and access into the LV is secured with the upwardly directed curve of the LV wire. The mitral valve and then interatrial septum predilation (5) are performed. Then, the mitral THV slow deployment (6) is performed. Both valves are deployed under rapid pacing using the LV wire. Abbreviations: LV, left vetnricle; STVR, simultaneous transcatheter valve replacement; TAVR, transcatheter aortic valve replacement; TEE, transesophageal echocardiography; THV, transcatheter heart valve; TMVR, transcatheter mitral valve replacement.

After withdrawal of the eSheath from the left femoral artery, a significant stenosis was seen in the TAVR primary access artery, which was successfully treated with balloon angioplasty using the same balloon (Figure 6). All other accesses were closed without complications, and the TAVR secondary access was closed with a 6-F Angio-seal VIP (Terumo, New Jersey, USA). All the steps are summarized in the step-by-step (Table 1 and Figure 7).

**Table 1:** Step-by-step guidance on performing a fully percutaneous transfemoral simultaneous transcatheter valve replacement (STVR) for native aortic regurgitation and mitral stenosis

Main steps	Step-by-step description	Remarks
1. Vascular accesses (Figure 3).	I. Primary venous access (TMVR) – Right femoral vein (6-Fr). II. Secondary arterial access – Right femoral artery (6-Fr) and cross-over technique to the left femoral artery. III. Primary arterial access (TAVR), contralateral left femoral artery (6-Fr, then upgrade for 9-Fr). IV. Half heparinization.	Ultrasound-guided vascular access. 300-cm GRAND SLAM® and a modified mammary catheter. Ultrasound- and cross-over-guided vascular access with preclosure technique.
2. TMVR setting (Figure 4).	I. Exchange venous access introducer for the VersaCross RF® system. - With the VersaCross Pigtail RF® wire II. Perform transeptal puncture. III. Advance and leave in place the introducer and wire into the LA. IV. Full heparinization	TEE-guided transeptal puncture: inferoposterior puncture. If no pericardial effusion.
3. TAVR procedure (Figure 4).	I. As per manufacturer recommendations, with an oversize of 20% and be ready to adjust while deploying under rapid pacing. II. Leave the TAVR wire in the LV. III. Leave the TAVR device introducer in place.	To secure LV access during and after the TMVR procedure.
4. TMVR procedure (Figure 5).	I. Exchange the VersaCross RF® introducer for an Agilis® NxT Steerable Introducer. II. Deliver TMVR wire into the LV through a pigtail catheter. III. Exchange Agilis® NxT Steerable Introducer for a GORE® DrySeal Flex (26-Fr). IV. Mitral valve and interatrial septum predilatation. V. TMVR valve delivery and deployment under rapid pacing. VI. Withdrawal of TMVR delivery system.	Under 3D to ensure safe passage into the LV. 14-16 mm balloon. Be prepared for THV postdilation. TEE evaluation, including LVOT obstruction and IASD.
5. Vascular closure (Figure 6).	I. Withdrawal and closure of primary venous access. II. Withdrawal and closure of primary arterial access with cross-over technique. - 1:1 balloon size selection for the left arterial iliac artery. III. Withdraw and closure of TAVR secondary access.	

Abbreviations: IASD, iatrogenic atrial septal defect; LA, left atrium; LV, left ventricle; LVOT, left ventricle outflow tract; TAVR, transcatheter aortic valve replacement; TMVR, transcatheter mitral valve replacement; TEE, transesophageal echocardiogram.

## Procedural and Short-Term Outcomes

The procedural transesophageal echocardiogram showed an aortic valve with a max gradient of 29 mmHg and a mean of 12 mmHg with no paravalvular leak. The mitral valve showed a max gradient of 15 mmHg and a mean gradient of 5 mmHg, with a trace of paravalvular leak with no LVOT obstruction. The patient was discharged home 24 ​hours later without complications. At the 1-month follow-up, the patient was asymptomatic in functional class I and preserved the same procedural results, with no criteria for obstruction or accelerations of the LVOT (LVOT max gradient pre 6.8 mmHg max gradient post 10 mmHg).

## Discussion

Fully percutaneous STVR for MVHD with a bi-femoral approach in concurrent AR and MS is feasible and effective in appropriately selected patients, mainly when performed by expert structural interventionalists. Nowadays, this approach should be considered in patients otherwise judged to have a very high or extreme surgical risk. Nevertheless, even with nondedicated THVs for both off-label indications, this double procedure effectively eliminated AR and MS without complications, including no LVOTO.

According to the Euro Heart Survey on VHD, MVHD, defined by at least 2 moderate VHDs, was observed in 20% of the patients with native VHD and 17% of those undergoing intervention.^15^ More recently and with clinical-meaningful definitions, the EURObservational Research Programme Valvular Heart Disease II Survey (EORP VHD II) presented characteristics, management, and outcomes of patients with native MVHD.^3^ Among 5087 patients with ≥1 severe left-sided native VHD, 70.2% had a single left-sided VHD, 7.1% had 1 severe left-sided VHD with moderate VHD of the other ipsilateral valve, and 22.7% had ≥2 severe native VHDs (left-sided and/or tricuspid regurgitation), with an overall prevalence of MVHD of 30%. Remarkably, patients with MVHD were often women with more significant hemodynamic impact (higher left atrial volumes, pulmonary pressures, and lower left ventricular ejection fraction). After valve interventions, they showed worse rates of heart failure and survival even after adjustment for baseline characteristics.^3^ Compared to single VHD patients, MVHD patients were more often left under medical treatment.^3^

Objective surgical risk estimation in this setting is challenging, and current validated tools do not account for simultaneous MVHD interventions.^2^ Although recent efforts to refine risk estimations on these patients are underway,^17^ overall surgical MVHD interventions carry higher morbidity and mortality risks than single VHD interventions,^17^ thus making less-invasive treatment options, such as catheter-based heart valve replacement interventions, an appealing alternative in selected cases.

In contrast to surgical double-valve replacement, for STVR, the aortic valve is replaced first. This allows the ventricle to have a lower pressure and enables it to handle the increased blood volume after mitral valve replacement for MS.^18^ In addition, since the aortic and mitral annuli are contiguous, bridged by the aortomitral fibrous curtain, some degree of obstruction could happen during the new aortic THV deployment if the mitral had been treated first.^19^

Although TAVR for AR is still an off-label indication, there is growing evidence of good procedural results and outcomes, particularly with new-generation valves.^20^ Most of the time, achieving approximately 20%^21^^,^^22^ of oversize is critical when using balloon-expandable non–AR-dedicated TAVR valves to pursue good sealing.^23^ Several challenges arise during THV deployment for AR: lack of calcifications of aortic annulus and cusps for the anchoring of the prosthesis, absence of fluoroscopic calcific landmarks, increased stroke volume, high back-and-forth flow status through the valve, aortic root dilatations, and annular eccentricity.^24^ Furthermore, migration into the LV or embolization into the aorta can occur up to several hours after implantation.^25^ Both balloon-expandable or self-expandable valves can be used for aortic regurgitation; however, there is more evidence behind balloon-expandable valves.^26^ Although the calcified sinotubular junction could have been a concern, the delivery balloon of a 23 mm S3 valve is 20 mm, and the simulated implanted height spared the calcified junction, so we decided to go with a balloon-expandable system.

Valve-in-MAC is one of the most challenging clinical scenarios of TMVR^27^ and, unfortunately, the one with the worst outcomes compared to valve-in-valve and valve-in-ring TMVR.^28^ Nevertheless, it is still a reasonable option for some highly selected patients.

Performing STVR for native MVHD is challenging in several aspects. It does not only imply the technical aspects of each transcatheter procedure by itself, but several additional considerations, like the stepwise vascular access setting, staged heparinization and maintenance of the LV wire after TAVR, are among the most important considerations.^29^ If using a SavvyWire (OpSens, Quebec, Canada) for the TAVR implantation, its invasive pressure measurement capabilities could be used to measure the resultant neo-LVOT gradients immediately after the TMVR.^30^

**Figure 8:**
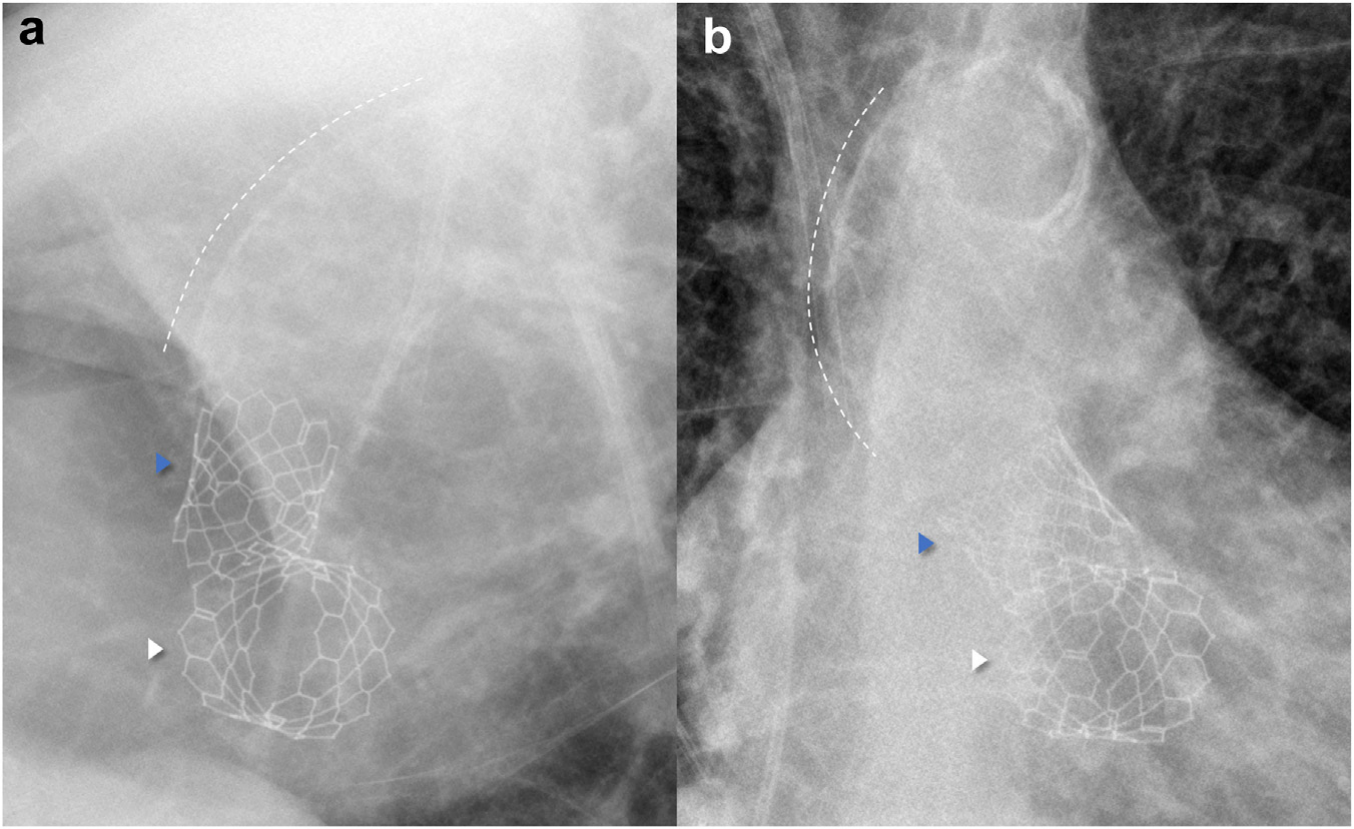
**Porcelain aorta, transcatheter aortic and mitral heart valves.** Lateral (a) and Anteroposterior (b) chest x-ray showing both SAPIEN S3 Ultra valves (Edwards Lifesciences, California, USA) in the aortic (blue arrowhead) and mitral (white arrowhead) positions, as well as the porcelain aorta (white dashed lines).

Simultaneous transcatheter valve replacement for native MVHD is far from being recommended as the standard of treatment for these patients. Factors that would make an STVR an unacceptable approach for these patients are mainly the presence of anatomical contraindications for TAVR or TMVR for valve-in-MAC. In the case of high-risk anatomical features such as borderline neo-LVOT, we would suggest having a lower threshold for not performing the STVR or performing it after successful septal ablation and/or leaflet modification techniques. Caution should be taken when performing this STVR approach, and the option of stopping the procedure at any time of the procedure should always be considered, particularly in cases where TAVR results are not as expected and mostly in inadvertent lower aortic valve implantation. After TAVR, the LVOT should be carefully evaluated to see if there were any changes from the baseline, which would suggest aborting the TMVR. Taking these precautions, the rationale behind this potential indication seems promising, and most importantly, it could be a viable treatment option for a large proportion of very high-risk patients and inoperable-judged patients left under medical therapy (Figure 8).

Although the STVR approach can increase procedural times (thus, longer exposure to heparin and intubation – if applicable) and the number of vascular accesses for a single procedure, it seems to be a reasonable approach when compared to a staged approach to avoiding exposing the patient to a second intervention and its procedural-related morbidity. Independently of the estimated surgical risk, TAVR procedures themselves are related to their own non-neglectable procedural-related mortality and complications. This is true also for TMVR procedures. The procedural time of this STVR was 94 ​minutes, which is similar to the average time of a single procedure and is shorter than the average total amount of time of a single TAVR and TMVR combined. Also, although it takes 3 vascular access sites, they do not represent more than the total amount of vascular accesses used for a staged approach. Additionally, whenever there were concerns about the TAVR results or an increase of gradients in the LVOT during the procedure, the procedure could be aborted before the TMVR phase without compromising the patient’s safety, allowing for evaluating different strategies in a staged manner. Nevertheless, with proper procedural planning in a non–high-risk LVOT and with good results with TAVR, no additional concerns are to be expected at this level.

This is the first case of fully percutaneous STVR for native MVHD in AR and MS (Table 2). STVR in this particular mixed valvular pathology is feasible and effective in expert hands with deep understanding and knowledge of both pathologies and their interactions, not only from a pathological point of view but also from the procedural aspects. Dedicated THVs, such as the Trilogy valve (JenaValve, California, USA) for AR^31^ or the vast myriad of dedicated mitral THVs,^32^ will definitively provide a more promising future for fully percutaneous STVR for selected patients with MVHD. Clinical studies are needed to study the results and outcomes of STVR techniques.

**Table 2:** Fully percutaneous transfemoral simultaneous transcatheter valve replacement (STVR) for multiple valvular heart disease (aortic and mitral)

Author	Aortic disease	Mitral disease	Aortic prosthesis	Mitral prosthesis	Replacement order	Complications
Bashir, et al.^12^	Aortic stenosis	MAC with mitral stenosis	SAPIEN S3: 23 mm	SAPIEN S3: 26 mm	A → M	No
Fanari, et al.^13^	Aortic stenosis	MAC with mitral stenosis	SAPIEN S3: 26 mm	SAPIEN S3: 29 mm	A → M	No
Mehta, et al.^14^	Aortic stenosis	MAC with mitral stenosis	Evolut PRO: 29 mm	SAPIEN S3: 29 mm	A → M	N/A

Abbreviations: A, aortic; M, mitral; MAC, mitral annular calcification; N/A, not available.

## Consent Statement

Consent was obtained for the procedure and scientific publishing of the anonymized case report.

## Funding

The authors have no funding to report.

## Disclosure Statement

The authors report no conflict of interest.

## Supplementary Material

Supplemental Video 1 STVR Step 1. Eco-guided transeptal puncture aiming posteroinferior.

Supplemental Video 2 STVR Step 2. Pre-TAVR aortogram showing severe aortic regurgitation.

Supplemental Video 3 STVR Step 3. Transcatheter aortic valve replacement under rapid pacing.

Supplemental Video 4 STVR Step 4. 3D eco-guided mitral valve crossing into the left ventricle.

Supplemental Video 5 STVR Step 5. Interatrial septum predilation after mitral valve predilation.

Supplemental Video 6 STVR Step 6A. Transcatheter mitral valve replacement under rapid pacing, moderate paravalvular leak.

Supplemental Video 7 STVR Step 6B. Post mitral valve implantation moderate paravalvular leak.

Supplemental Video 8 STVR Step 6C. TMVR postdilation with 3 ml extra added to the balloon.

Supplemental Video 9 STVR Step 6D. Final trace of paravalvular leak.

Supplemental Video 10 STVR Immediate and follow-up results. Immediate transmitral and transaortic gradients and follow-up neo-left ventricle outflow tract gradients.
